# Subcutaneous Fat Necrosis and Hypercalcemia After Therapeutic Hypothermia in Patients With Hypoxic-ischemic Encephalopathy: A Case Series

**DOI:** 10.7759/cureus.3074

**Published:** 2018-07-30

**Authors:** Sourabh Verma, Sean M Bailey, Pradeep V Mally, Elena V Wachtel

**Affiliations:** 1 Division of Neonatology, Department of Pediatrics, New York University School of Medicine, New York, USA; 2 Division of Neonatology, Department of Pediatrics, New York University School of Medicine, New York , USA

**Keywords:** subcutaneous fat necrosis, therapeutic hypothermia, hypercalcemia, hypoxic ischemic encephalopathy, newborns

## Abstract

Therapeutic hypothermia (TH) is provided to newborns with moderate to severe hypoxic-ischemic encephalopathy (HIE) to improve survival and long-term neurodevelopmental outcomes. Although the benefits certainly outweigh the risks associated with therapeutic hypothermia, it is important to be mindful of potential rare side effects in the background of asphyxia-related injury to various body organs. One of those side effects includes subcutaneous fat necrosis (SCFN) that can occur in term newborns after perinatal hypoxia-ischemia or other stressing factors such as systemic hypothermia. It is usually a self-limited condition, however, in some cases, it can lead to severe hypercalcemia. We report three such cases of SCFN in newborns with HIE treated with TH. Due to potential long-term complications, such as metastatic calcifications, caregivers should be informed about this potential complication prior to discharge from hospital so that they can help diagnose or continue to monitor cases of severe hypercalcemia.

## Introduction

Perinatal asphyxia can cause hypoxic injury to various body organs such as the brain, heart, liver, intestines, and skin. Therapeutic hypothermia (TH) has become the standard of care for newborn infants with moderate to severe hypoxic-ischemic encephalopathy (HIE) and has been shown to improve survival and neurodevelopmental outcomes [1]. Some of the transient adverse effects commonly associated with TH in the background of asphyxia include bradycardia, hypotension, and prolonged coagulation profiles [1]. All of these warrant close evaluation and monitoring. Another rare side effect of TH that has been reported in the literature as occurring in approximately 1% of newborns with perinatal asphyxia is subcutaneous fat necrosis (SCFN) [2-4]. SCFN is characterized by indurated nodules or plaques, with or without erythema, that usually appear within the first few weeks of life [5-6] over bony prominences, such as the back, buttocks, scalp, legs, and arms. Usually, this pathology resolves over time without any treatment. However, it may be complicated by the presence of other side effects such as high serum calcium levels [7-8]. Hypercalcemia in these infants may become symptomatic and require treatment in a few cases with hydration, loop diuretics, glucocorticoids, and pamidronate. Other complications of SCFN such as hyperglycemia, hypoglycemia, hypertriglyceridemia, and thrombocytopenia have been reported, but their exact association with TH remains controversial [2,9].

In this case series, we report three cases of SCFN in infants admitted to our Bellevue Hospital regional perinatal center (RPC) with perinatal asphyxia. Our RPC is a busy referral center, catering to the needs of 11 New York City (NYC) health + hospitals institutions in the NYC metropolitan area. We provide therapeutic hypothermia treatment to approximately 20-25 infants with HIE every year.

## Case presentation

The following are the three case reviews of infants with SCFN after receiving TH; all of them developed hypercalcemia and one of them required treatment with pamidronate. 

Case 1

**Figure 1:**
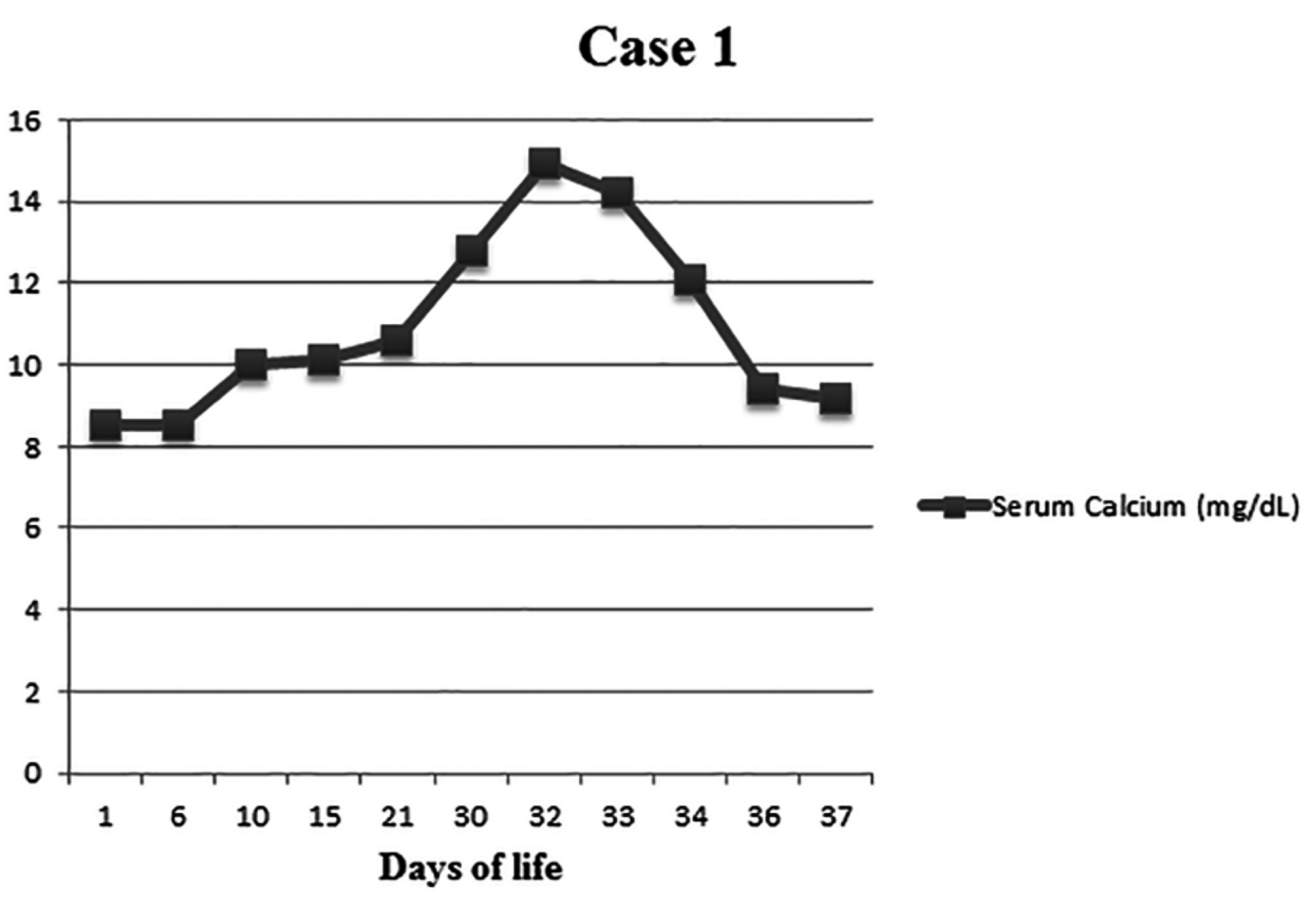
Serum calcium levels (mg/dL) in the context of days of life for patient case 1

An early term female infant born at the 37^+0^ weeks gestation age, of 3126-gram birth weight, was delivered via stat cesarean section for decreased fetal movements and fetal bradycardia. The infant was born limp, pale, and required resuscitation with positive pressure ventilation, intubation, chest compression, epinephrine, and normal saline boluses. Apgar (appearance, pulse, grimace, activity, respiration) scores were 1, 1, 2, and 3 at 1, 5, 10, and 15 minutes of life, respectively. Initial umbilical cord venous blood gas had a pH of 6.86 with a base deficit of 15.6 mmol/L. The baby was started on antibiotics and transferred to our hospital for therapeutic hypothermia. The baby developed episodes of extension of arms and legs consistent with a seizure. Phenobarbital was loaded intravenously with a resolution of seizures. The neurological exam and amplitude-integrated electroencephalography (aEEG) tracing were suggestive of severe HIE. The baby was started on whole body cooling, which continued for 72 hours. The baby had hypoxic respiratory failure and was started on nitric oxide for the management of persistent pulmonary hypertension confirmed on echocardiography. Video-electroencephalography (vEEG) done on day of life (DOL) 6 was significant for profound diffuse cerebral dysfunction. Magnetic resonance imaging (MRI) brain showed diffuse restricted diffusion of the supratentorial cortex with preservation of infratentorial structures, suggestive of significant HIE. On DOL 10, the baby was noted to have slightly erythematous, indurated nodules on the upper back, posterior axilla, sacrum, and buttocks. The pediatric dermatology team was consulted, which diagnosed these lesions as subcutaneous fat necrosis, and suggested no treatment at that time with the monitoring of serum or ionized calcium (iCal) looking for hypercalcemia. The serum calcium level was noted to be higher at 10.6 mg/dL (iCal: 6.07 mg/dL) on DOL 21 and pediatric endocrinology was consulted. Parathyroid hormone levels were sent to rule out hyperparathyroidism as a cause of hypercalcemia and came adequately suppressed. The baby was initially given furosemide and adequate hydration in view of the increasing calcium levels. The serum calcium level continued to rise with a peak of 14.9 mg/dL (iCal: 7.5 mg/dL) on DOL 32. The baby did not have any other signs and symptoms of hypercalcemia such as electrocardiographic changes, vomiting, and hypertension. Since levels continued to increase despite hydration and diuretic therapy, the baby was started on hydrocortisone and given a dose of pamidronate. This helped in decreasing calcium levels, which eventually came back to the normal range on DOL 35 (Figure 1). The baby was discharged home on a low calcium formula with a resolution of skin lesions. On follow-up visits, the baby had normal serum calcium levels and no new skin lesions.

Case 2

**Figure 2:**
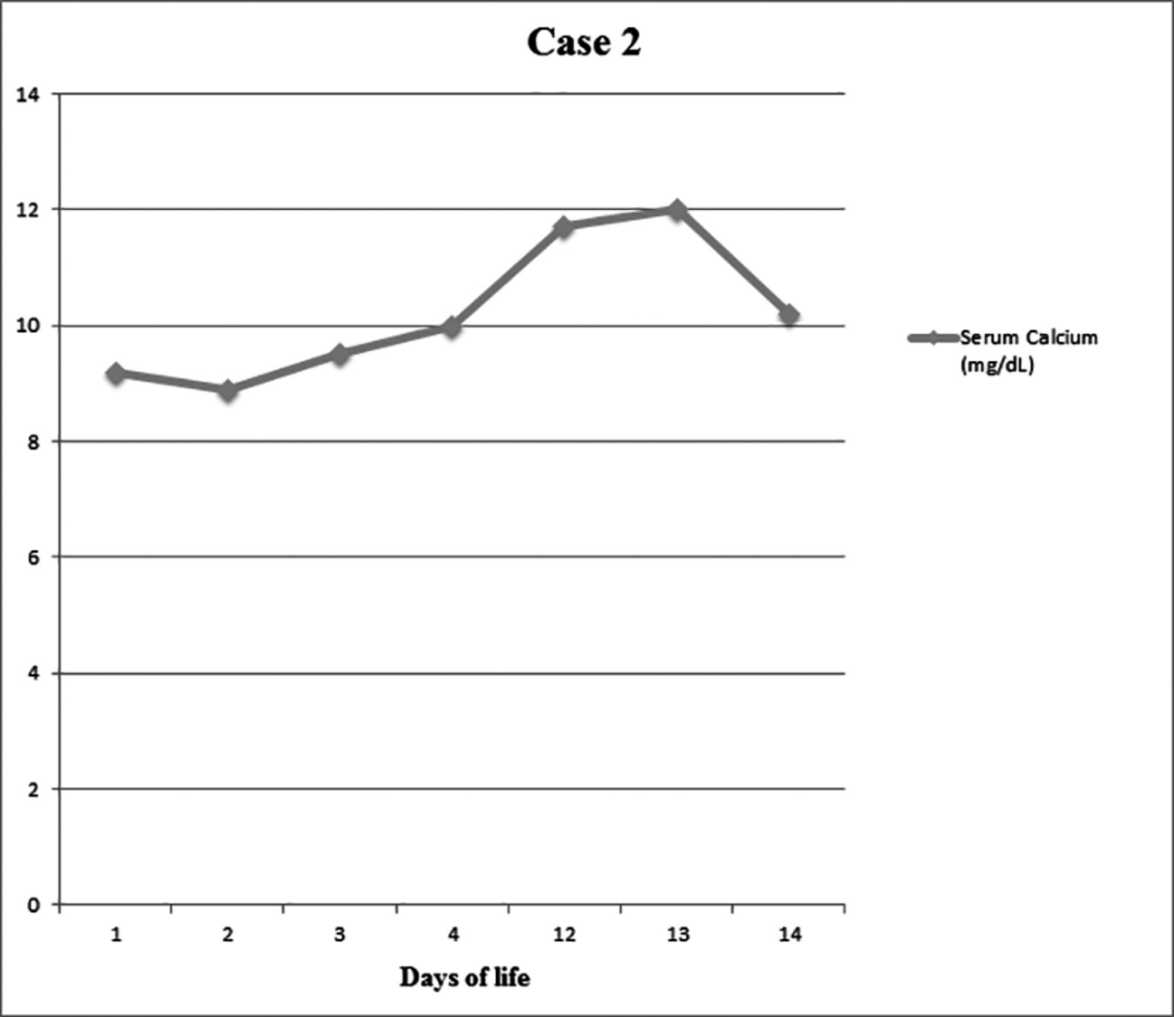
Serum calcium levels (mg/dL) in the context of days of life for patient case 2

A full-term male infant born at 39^+5^ weeks gestation age, of 3265 gram birth weight, delivered via urgent cesarean section for absent fetal movements, decreased variability, decelerations, and thick meconium-stained amniotic fluid. The infant was born limp, pale, and was intubated at delivery. Apgar scores were 1, 6, and 7 at 1, 5, and 10 minutes of life, respectively. The baby had severe metabolic and lactic acidosis (umbilical venous cord gas with pH 6.99, pCO2 128 mm of Hg, base deficit 10 mmol/L, and lactic acid 20 mmol/L), and moderate hypotonia on examination. After initial resuscitation and stabilization, the baby was transferred to our RPC for therapeutic hypothermia. The baby was evaluated and demonstrated the signs and symptoms of moderate HIE. The infant met the criteria for whole body cooling. The baby was transitioned to continuous positive airway pressure (CPAP) ventilation on DOL 1 and to room air on DOL 13. Troponin levels went down from 0.296 ng/mL on DOL 1 to 0.025 ng/ mL on DOL 3 and never required vasopressors during the hospital stay. The baby received two transfusions of fresh frozen plasma to correct abnormal coagulation profiles as per our hypothermia protocol. Video EEG performed on DOL 5 showed no seizure activity or dysmature, discontinuous sleep pattern for gestation age. MRI brain showed no apparent signs of HIE and a small focal periventricular acute infarct in the right temporal-occipital junction. On DOL 12, the infant was noted to have firm, ill-defined subcutaneous plaques and nodules with subtle overlying erythema at the middle of the back, bilateral posterior scapula, and left areola with the expression of white fluid with pressure on the nipple. Pediatric dermatology was consulted, which diagnosed the lesions as SCFN, with possibly a liquefactive necrosis of lesion in the left areolar area. Daily calcium levels were checked, which showed a peak serum level of 12.7 mg/dL on DOL 13 with subsequent resolution, adequate hydration, and a dose of furosemide only (Figure 2). The baby was given follow-up sub-specialty appointments with pediatric dermatology, endocrinology, and nephrology. The patient was discharged home on a low calcium formula with a weekly monitoring of calcium levels.

Case 3

**Figure 3:**
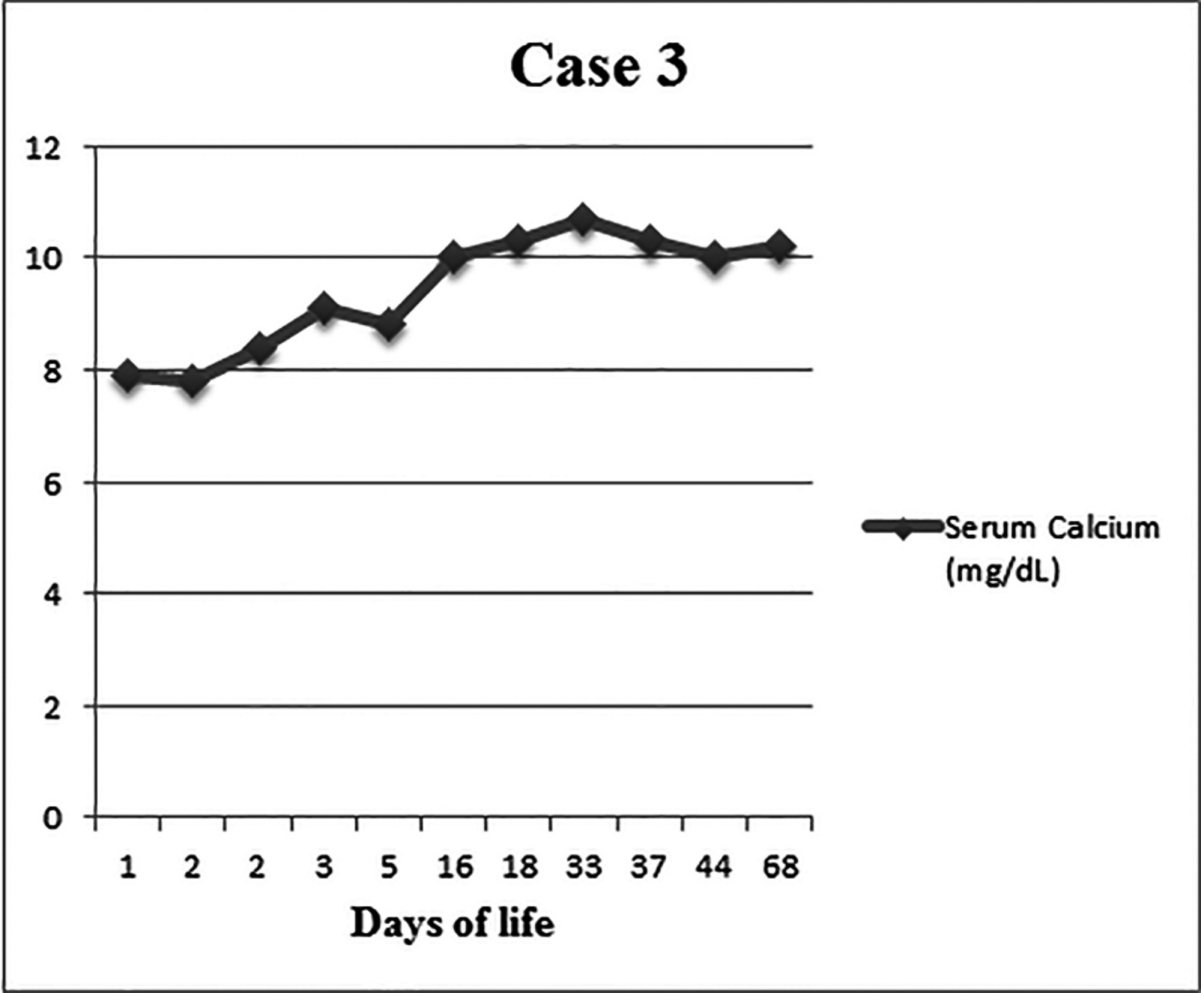
Serum calcium levels (in mg/dL) and days of life

A full-term female infant born at 39^+0 ^weeks of gestation, of 2750 gram birth weight, was delivered at the referring facility by an urgent cesarean section for fetal decelerations and abruptio placentae. The infant was born limp with no respiratory efforts. Cardiorespiratory resuscitation was performed, which involved endotracheal intubation, chest compressions, and intravenous epinephrine administration. Apgar scores were 1, 1, 1, and 4 at 1, 5, 10, and 15 minutes of life, respectively. The infant had severe metabolic acidosis (umbilical arterial cord blood gas pH 6.72 with a base deficit of 26 mmol/L, the initial blood gas pH in the neonatal intensive care unit was 6.80, with a base deficit of 25 mmol/L and lactic acid 19 mmol/L) and an abnormal neurological examination. The baby was transferred to our RPC for therapeutic hypothermia. The infant had pulmonary hypertension on echocardiogram and was placed on a high-frequency oscillator and inhaled nitric oxide. The infant was extubated on DOL 5 and transitioned to room air on DOL 15. The patient was on vasopressors for hypotension during the first three days of life. MRI brain showed bilateral parieto-occipital infarcts involving the splenium and thalamus consistent with HIE. Video EEG showed subclinical seizures. On DOL 15, multiple erythematous indurated nodules were noted on the occipital scalp. The pediatric dermatology service was consulted and lesions were diagnosed as SCFN. Serum calcium levels at the time of presentation were 10.0 mg/dL. The patient was discharged home on DOL 21. Scalp lesions were resolved by DOL 25. Peak serum calcium levels were 10.7 mg/dL at DOL 32 at an outpatient follow-up visit. Serum calcium levels were normalized by DOL 39 (Figure 3).

## Discussion

SCFN is a rare, but possibly serious, side effect seen in infants with HIE and treated with TH because of its potential to lead to severe hypercalcemia. It is a non-infectious panniculitis that involves the necrosis of granulomatous fat cells in subcutaneous tissue. Fat cells necrosis may promote the release of active Vitamin D, which then leads to the increased absorption of calcium and hypercalcemia.

**Figure 4:**
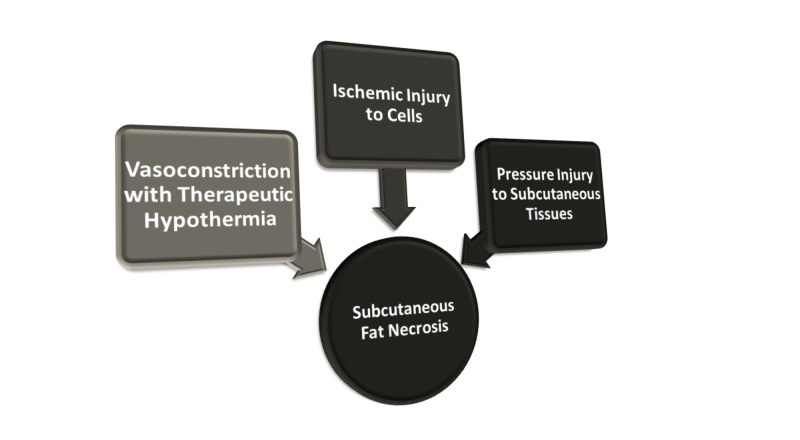
Etiology of subcutaneous fat necrosis in infants receiving therapeutic hypothermia for hypoxic-ischemic encephalopathy

Various factors may play a role in the development of SCFN [3,10], such as decreased perfusion of subcutaneous tissues as a result of hypoxic injury at birth. Other possible risk factors for SCFN have been described such as hypoglycemia, anemia, thrombocytosis, and systemic hypothermia. A stressful reaction to a cold surface area and less frequent repositioning of infants while receiving TH can further place already under-perfused tissue to the risk of pressure-induced traumatic injury (Figure 4).

Since whole-body cooling is being employed more frequently at therapeutic hypothermia centers, this may potentially put these infants at increased risk for SCFN in comparison to selective head cooling. Limited knowledge of the rare side effects of perinatal asphyxia and systemic hypothermia, such as SCFN, accompanied by an incomplete physical examination, especially at the back, buttocks, and posterior aspect of the scalp and extremities, can lead to missed or delayed diagnosis. Early Vitamin D and calcium supplementation after receiving TH without measuring serial serum calcium levels and carefully evaluating the presence of any skin lesions may predispose at-risk infants to hypercalcemia.

Most of these nodules or plaques may resolve over a period of time. But those infants with SCFN, whose calcium levels become very high or if they become symptomatic with hypercalcemia, should be considered to receive treatment. In less severe cases, dietary restriction of calcium (by using formulas with low calcium concentration) and vitamin D (avoiding supplementation) may help. Other therapeutic options include maintaining hydration, using calciuric loop diuretics, such as furosemide, glucocorticoids, and bisphosphonates, such as pamidronate. The management requires a multidisciplinary approach with various subspecialties such as pediatric dermatology, endocrinology, and nephrology.

Hypercalcemia may also cause calcification in the genitourinary system. Therefore, it may be useful to obtain a whole abdomen sonogram to rule out nephrocalcinosis. After conducting a literature search, we found one case of myocardial calcification as a complication of SCFN as well [11]. During TH, frequent repositioning of the infants to avoid any further pressure-induced traumatic injury to already susceptible and underperfused subcutaneous tissue may be helpful. Consider also giving sedation during TH, as this may help some patients. Careful daily physical examinations to look for any indurated skin areas and serial calcium measurements even after finishing TH may also help in early diagnosis and management.

**Figure 5:**
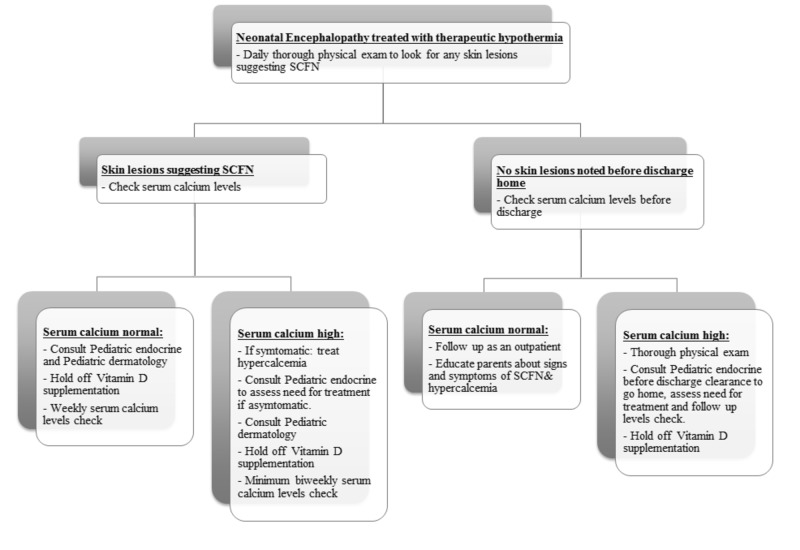
Suggested diagnostic and management algorithm for subcutaneous fat necrosis

Since signs of SCFN may develop days to weeks later, following perinatal asphyxia and TH, there remains a chance of developing these skin lesions after being discharged from the hospital. Therefore, it is important for pediatricians to know this potential complication of TH, the signs to look for, and appropriate management. Also, checking serial serum calcium levels until two months of life in high-risk infants may help in diagnosing hypercalcemia early on. Similarly, it may be useful to educate parents about the features of skin lesions associated with SCFN and the signs of hypercalcemia and other late but rare complications of HIE and TH. We have proposed a flow diagram for neonatologists and pediatricians to help in managing cases with SCFN (Figure 5).

## Conclusions

We report three cases of SCFN in newborns with HIE treated with therapeutic hypothermia at our RPC. TH is the standard of care for patients with HIE. It is prudent to closely monitor some of the rare side effects in patients with HIE receiving TH, such as SCFN. It usually gets resolved in the first few days of life but, in some cases, may get associated with serious side effects, such as hypercalcemia. Since these serious side effects may occur days to weeks after completing therapeutic hypothermia, it is important to measure calcium levels at the time of discharge from the hospital. Primary pediatricians taking care of these infants after discharge from the hospital should monitor the calcium levels as an outpatient. Parents should be educated to look for any skin lesions and signs of hypercalcemia at home so that they could bring their child for evaluation in a timely manner. More research is needed to study various sub-factors associated with SCFN and its prevention. Future studies may help in establishing guidelines for the diagnosis and management of SCFN in babies receiving TH after HIE.
